# First Annual Meeting of the Dental Association of Western New York

**Published:** 1863-11

**Authors:** Leon F. Harvey


### Proceedings of Societies.

---

### FIRST ANNUAL MEETING OF THE DENTAL ASSOCIATION OF WESTERN NEW YORK.

REPORTED BY LEON F. HARVEY, M. D.

This Society held its first annual meeting in Buffalo, on Tuesday, Oct. 6th, 1863.

The President, C. H. Harvey, M. D., in the chair. The minutes as read by the Secretary were adopted.

Dr. Leon F. Harvey, on motion, was invited to report the proceedings for the Journals.

The election of officers for the ensuing year resulted as follows:

*President*—Dr. B. T. Whitney, of Buffalo.

*Vice-President*—Dr. L. W. Bristol, of Lockport.

*Secretary*—Dr. Geo. Field, of Rochester.

*Treasurer*—Dr. E. L. Wood, of Brockport.

The newly elected officers were conducted to their respective chairs, by Drs. Walter and Snow.

The President, Dr. Whitney, thanked the Society for the honor conferred upon him. Said that he would endeavor to do his duty, and hoped to be aided by the members. Much depended upon the chair, and very much upon the members for the interest of the meetings.

Dr. LEON F. HARVEY read an essay entitled Epulis. On motion, a vote of thanks was given Dr. Harvey for his able paper, and a copy requested for publication.

FILLING TEETH.

Dr. G. B. SNOW is using cylinders more and more—uses adhesive gold with them. Likes Wood's Plastic Metallic Filling. Prefers it to amalgam.

Dr. BRISTOL thinks nothing is gained over the amalgam by it. Anneals his foil by placing it in a platinum cup, and heating till all the animal matter is burned off, which he finds out by holding his face over the cup till no smell is noticed.

Dr. R. G. SNOW, uses cylinders a little longer than the depth of the cavity; crowds them home with a heavy instrument—fills in between with adhesive foil.

Dr. WALTER, hoops teeth with silver when but one wall is remaining; uses cylinders, pellets and adhesive foil—bevels the edges of the cavity to have the filling sustain the walls. Prefers to drill a hole through the enamel to hold the gold; heats his gold till it begins to change color. When the cavity is partially filled and the gold becomes wet, use chloroform in it, and then dry out with paper; use adhesive foil—the chloroform removes the grease.

Dr. B. T. WHITNEY, in filling of upper bicuspids, anterior and posterior approximal surfaces, opposite each other, did not make a crown cavity as many do; but cut away freely, making the opening V shaped. Uses cylinders and gold folded upon itself a number of times—having filled part way up, uses annealed gold. Places a wedge of wood between the teeth near the gum; is not troubled with bleeding, if the gum is touched. No saliva; thinks Wood's metal good, depends a great deal on the instruments used.

Dr. BRISTOL, not an advocate of arsenic; prefers to extract teeth with exposed nerves; loses eight out of ten that he fills.

Dr. R. G. Snow, saves eight out of ten, uses arsenic—does not allow it to remain in longer than twenty-four hours; in three or four days after removing the arsenic, takes out the dead nerve; fills in ten to fourteen days after the removal of the nerve. If tooth becomes in a few months, sore, passes a current of electricity through it for ten or fifteen minutes, and the pain is abated.

Dr. Hamilton, removes the arsenic in ten or twelve hours; finds that the bone of the tooth has become inflamed, removes the inflammation by the third attenuation of creosote.

Dr. Geo. B. Snow here read an essay, entitled, "The Treatment of the Dental Pulp."

On motion, a vote of thanks was offered Dr. Snow for his able and interesting paper, and a copy was requested for publication.

Dr. Walter, is very guarded in the use of arsenic, especially in cases of scrofula and rheumatism, and in persons in whom wounds inflame readily. Thinks that creosote increases the inflammation due to arsenic; has found Tinct. Ginger to act well as an antiphlogistic.

Dr. Bristol, uses for broaches to extract nerves, very fine ragged strips of gold or silver.

Dr. G. B. Snow says that the best way to anneal broaches is to place them in a tube filled with charcoal, and heat it red hot.

Dr. B. S. Brown, makes his broaches from piano forte wire.

On motion, Dr. Taft, of Cincinnati, was made an honorary member.

Order of business was here suspended to allow the society to appoint delegates to the American Dental Association next year.

Dr. Taft being called upon gave some of the benefits to be derived from a central Association. Encourages local ones; collecting from all, can find the best method for conducting a society. The Central depends on local socities for its existence. Within the last year five new societies have been organized.

Committee appointed to choose delegates submitted the following names:

C. H. Harvey, B. T. Whitney, R. G. Snow, L. D. Walter, J. Requa, J. Harrmon, E. L. Wood, J. L. Clarke, O. F. Roffee, J. G. Barbour, L. W. Bristol, and V. A. Lord.

Dr. J. Lewis, agrees with Dr. Bristol, that there is very little use to save a tooth with an exposed nerve. When pulp is inflamed uses a weak solution of camphor (then increasing the strength) to the gums, being very careful not to touch the neck of the tooth, which will increase the inflammation. Having reduced the inflammation, cap the nerve with silver, gold will destroy the pulp. Physicians know that in trepanning, gold can not be used to cap the opening; leave as much decay over the pulp as possible—has saved more teeth in this way, than when the nerve has been destroyed.

Dr. Whitney uses arsenic in his practice; removes inflammation by applying sulphuric ether to the gums.

Dr. Taft,—A tooth does not depend entirely on the pulp for its vitality, but on that and the periosteum; dead teeth decay sooner than living ones. In a case of an exposed nerve many things are to be taken into consideration,—how long the pulp has been exposed; how large the orifice; the constitution of the patient. If not exposed long, and the opening is not large, and the constitution is good, cover with cotton, spunk or Hill's stopping; creosote being applied, and fill with gold, being careful not to touch the sensitive point.

If there is inflammation, treat constitutionally, give Rochelle salts; remove local irritation by removing the cause, close up the cavity with cotton saturated with creosote; get the parts in a normal condition. Sometimes it is difficult to destroy the pulp; applied arsenic six times in as many days, and yet the pulp remained alive. Prefers to remove the pulp by a surgical operation; is best in every sense; there is no risk as with a therapeutic agent. In operating on the anterior teeth, there is little or no pain or difficulty; always makes as direct entrance into the canal as possible, through the palatine surfaces; make the opening large; nerve extractor must be perfectly smooth; make a curve in it by placing the point on an anvil and making a turn. In carrying it into the canal run the edge against the wall, and curved portion against the pulp, turn it suddenly and excise the pulp; it gives no pain, unless there is a ragged edge. With molar teeth pursues a different course; mixes arsenite of cobalt and charcoal equal parts together, and a little creosote—finds that the arsenite of cobalt is not taken up as arsenious acid. Often removes by excision from the molars, but it is very difficult; removes sometimes the pulp and nerve from the palatine fang, and allows the others to remain. Thinks that they generally die, but sometimes live; ossification takes place sooner in buccal than palatine fangs, the debris usually remaining is not enough in these cases to produce difficulty.

Dr. Bristol asked Dr. Taft if he could do any thing if the tooth should become sore.

Dr. Taft, always tells patients that they *may* become sore. If an inflammatory diathesis exists, treat constitutionally; give salts. If hands or feet are cold, equalize the circulation by warm baths; if there is an unusual flow of blood to the part, sometimes may apply a leech—counter-irritation.

Two years ago filled for a lady the second left superior bicuspid; most of the crown gone, inner portion standing, pulp dead; built the crown up with gold. Last fall filled for her the second inferior right bicuspid; wanted at that time the upper tooth out. Thursday, she felt some pain; Saturday, she said she must have it out—Friday the pain stopped, probably due to some periodical or accidental discharge. In May, her husband said I must go and see his wife, as she was suffering very much. Supposed it was perostitis, and armed myself with leeches, lancet, forceps, etc. She had suffered the day previous perfect agony with the under tooth. Enquired the cause, found that the menstrual discharge had been checked by being on the wet pavement; applied foot baths, used leeches and the lancet, but no good resulted; ordered something to induce the menstrual flow. Monday at nine, there was a slight discharge and the pain ceased, though the soreness continued till the next period. These functions are connected with the teeth more than dentists seem to think or acknowledge. During gestation many teeth are lost that ought to be saved. Finds no difficulty in approaching patients on these subjects; knows of nothing better to reduce local inflammation in some cases than sulphuric ether; for inflammation in deciduous teeth uses alum triturated in sulphuric ether.

Dr. WHITNEY, has removed very severe pain by the application of sulphuric ether to the gum and face; has no hesitation in addressing patients in regard to the menstrual period, when he has reason to suspect that it is the cause of pain.

Dr. GEO. B. SNOW, in filling follows up all of the crevices, leaves no discoloration.

Dr. WHITNEY illustrated his method of filling bicuspids (reported elsewhere), on the black board.

Dr. TAFT spoke of the necessity of concentrating the mind upon one subject; suggests that operators talk or think too much upon other subjects while filling. Does not allow himself to be disturbed while operating; not only the eyes, but the mind must be fixed upon the work. Dentists must have a uniform temper, never be irritated; if a difficult operation is to be performed, prepare yourself for it—be careful about eating, and have plenty of sleep. The hand must be trained; hold the instrument as you would a pen; instruments should be as straight as possible.

Canandaigua was chosen as the next place of meeting,—first Tuesday in May, 1864.

Dr. Whitney advised the preparing of a history of dentistry in Western New York.

On motion, a committee of five, consisting of Drs. R. G. Snow, L. F. Harvey, E. H. Roffee, L. W. Bristol and J. Requa, was appointed to collect material for such a work.

Dr. Whitney announced the death of Dr. O. B. Fellows, of Albion, who was present at the organization of the Association.

It was then moved that the meeting be adjourned till two o'clock.

### ALVEOLAR ABSCESS.

Dr. Taft,—It is supposed by many when an abscess occurs it is useless to keep a tooth, which is very wrong. Occurs generally from local irritation; all important to note the condition of the system. The most usual cause is a dead tooth, together with an acrid condition of the decomposed pulp, and excretion therefrom. Calcareous material upon the root by some is supposed a cause. Drill out the canal, wash it out with chloride of soda and water; if pus at the end of the fang, it is preferable to treat through the gums. Sometimes it is well to fill the fang, and then treat through the gum; it is well to find where the sac is, probe will reach it; destroy its ability to secrete pus—may cut it off; creosote or Per sul. Iron destroys it in a short time. If inflammation should occur after the breaking up of the sac, resort to depletion in a day or two after the removal of the sac. May use a leech or the lancet, creosote and iodine, tannin and iodine, creosote and tannin; there is often considerable destruction of bone in abscess.

Three years ago a young man who had suffered much with chills, came to me to have his teeth examined; his six anterior teeth were so loose, that they could have been picked out with the fingers. There was an abscess on each one of them, and an opening from each tooth—treated him constitutionally, used tonics. Removed pieces of bone, washed the passages out and used creosote, at first diluted, then glycerine and tannin; soon the inflammation subsided. Two months after filled all his teeth, and in three months discharged him cured;—there is a growth in the process. Saw him a few days ago, and all his teeth are firm. Dead teeth that are firm, do not occasion irritation.

Dr. R. G. Snow presented a canine tooth, extracted from the mouth of a lady who said that she had had all her teeth, and they were extracted long ago.

Dr. Whitney presented a tooth which twenty-three years ago was extracted and filled, then placed in the socket; a short time since he extracted it. Also showed a case of building a plate out with rubber, to give fullness to the cheeks.

Dr. Brown, of Canada, gave his method of covering, in vulcanite work; the inside of a plate to have the surface smooth. He places heavy tin foil upon the gutta percha and wax, it being ready to harden, cuts the edges and folds them nearly on to itself; then pouring the plaster in, when hard, the tin foil comes out with the teeth, and you will then have a perfectly smooth surface.

Dr. Bristol, uses quick lime as the best thing for polishing rubber.

Some remarks were made on the use of rubber and continuous gum together.

The Committee on subjects for discussion for next meeting, submitted the following:

Management of deciduous teeth. Hemorrhage after extraction. Extraction of teeth. Anæsthetics. Discoloration of teeth and its removal. Treatment of sensitive dentine. Flling teeth, best materials for the purpose. Methods of operating in particular cases. Treatment of alveolar abscess. Mechanical dentistry, including the best method of taking impressions.

Dr. Everett uses gutta percha for taking impressions; considers it the best material in use.

It being decided to have a seal procured for the society, Dr. Brown, of Canada, kindly offered to cut one, and present it to the society.

The President thanked him for his offer.

Dr. Taft made some remarks on rubber and the education of the people.

Dr. W. G. Oliver moved a vote of thanks to the officers of last year. Carried.

On motion, the society adjourned to meet at Canandaigua.

